# Efficacy of ginkgo biloba extract in the treatment of idiopathic pulmonary fibrosis: a systematic review and meta-analysis of randomized controlled trials

**DOI:** 10.3389/fphar.2025.1524505

**Published:** 2025-03-05

**Authors:** Xuxin Sun, Ling Peng, Wenchao Xiao, Keying Li, Sheng Chen

**Affiliations:** ^1^ The Fourth Clinical Medical College of Guangzhou University of Chinese Medicine, Shenzhen, China; ^2^ Shenzhen Traditional Chinese Medicine Hospital, Shenzhen, China

**Keywords:** Ginkgo biloba extract, idiopathic pulmonary fibrosis, meta-analysis, systematic review, RCT, randomized controlled trial

## Abstract

**Objective:**

This systematic review and meta-analysis aims to assess the efficacy of GBE in the treatment of IPF by evaluating its impact on total effective rate, blood gas analysis, pulmonary function tests, and markers of inflammation and fibrosis.

**Methods:**

We conducted a comprehensive search across seven databases, including PubMed, EMBASE, Web of Science, CNKI, Wanfang DATA, VIP, and CBM, without restrictions on publication date. Randomized controlled trials (RCTs) that investigated the effects of GBE on IPF patients were eligible for inclusion. Relevant literature was screened, and the data in the included studies were extracted for quality assessment according to the Risk of bias tool.

**Results:**

A total of 14 RCTs involving 1043 patients were included in the analysis. GBE significantly improved the total effective rate, arterial oxygen partial pressure, arterial oxygen saturation, forced vital capacity, forced expiratory volume in one second, maximum voluntary ventilation, and 6-min walk test compared to the control group. Additionally, there was a significant reduction in arterial carbon dioxide partial pressure, interleukin-4, hyaluronan, and laminin levels.

**Conclusion:**

GBE may offer therapeutic benefits in IPF by improving respiratory function, modulating inflammation, and affecting fibrosis markers. These findings support the potential use of GBE as an adjunct therapy in IPF and suggest that further large-scale, multicenter trials are warranted to confirm its efficacy and safety.

## 1 Introduction

Idiopathic Pulmonary Fibrosis (IPF) is a devastating interstitial lung disease characterized by the progressive replacement of functional lung tissue with fibrotic scar tissue, leading to impaired gas exchange and ultimately respiratory failure (Raghu et al., 2022). The etiology of IPF remains obscure, and despite recent advances in understanding its pathogenesis, the disease continues to carry a poor prognosis, with a median survival time of only three to 5 years following diagnosis (Liu et al., 2022a). The complexity of IPF’s pathogenesis, which encompasses chronic inflammation, dysregulated wound healing, and microenvironmental changes that drive fibroblast proliferation and extracellular matrix deposition, poses significant challenges for disease management (Mei et al., 2021).

Current pharmacotherapeutic options for IPF are limited, primarily focusing on slowing disease progression and alleviating symptoms. Anti-fibrotic agents, such as pirfenidone and nintedanib, have demonstrated modest benefits in slowing the decline in lung function (Flaherty et al., 2019; Finnerty et al., 2021). However, these treatments are associated with significant side effects and high costs, highlighting the need for alternative or complementary therapeutic strategies.

Ginkgo biloba L. extract (GBE) is a mixture with various pharmacological effects extracted and processed from Ginkgo biloba leaves, and its main active ingredients are 22%–27% flavonoids (quercetin, etc*.*), 5%–7% terpene lactones (ginkgolides, bilobalide, etc.), organic acids and phenols (Kulić et al., 2022). The oral formulation contains 19.2 mg of total flavonoid glycoside and 4.8 mg of terpenoid lactone or 9.6 mg of total flavonoid glycoside and 2.4 mg of terpenoid lactone. Recently, it has gained scientific attention for its potential anti-inflammatory, antioxidant, and vasorelaxant properties (Hu et al., 2024; Liu et al., 2024a). Clinical trials have explored GBE’s efficacy in enhancing memory, attention, and other cognitive domains, with some studies indicating a positive impact in both healthy individuals and those with mild cognitive impairment (Xiao et al., 2024). Beyond cognitive enhancement, GBE’s antioxidant and vasodilatory properties have prompted investigations into its potential role in treating cardiovascular diseases (Liu et al., 2024b). Furthermore, its anti-inflammatory and antioxidant effects have led to studies examining the use of GBE in respiratory conditions, such as IPF. Preclinical studies suggest that GBE may modulate key pathways involved in IPF pathogenesis, including the inhibition of pro-inflammatory cytokines, reduction of oxidative stress, and improvement of endothelial function (Zhu and Liu, 2024; Nie et al., 2024). These findings have spurred clinical investigations into the potential therapeutic benefits of GBE for patients with IPF.

Despite the growing body of literature on the use of GBE in IPF, there is a lack of consensus regarding its efficacy and safety (Yao et al., 2024). A systematic review and meta-analysis of the available evidence is necessary to synthesize the results of these studies and provide a comprehensive assessment of the potential benefits and risks associated with GBE treatment in IPF patients.

## 2 Methods

The protocol for this systematic review and meta-analysis has been registered with the International Prospective Register of Systematic Reviews (PROSPERO) under the identifier CRD42024603534. We adhered to the Preferred Reporting Items for Systematic Reviews and Meta-Analyses (PRISMA) guidelines for reporting (Moher et al., 2015).

### 2.1 Search strategy

We conducted a comprehensive literature search across seven databases: PubMed, EMBASE, Web of Science, CNKI, Wanfang DATA, VIP, and CBM. The search was not restricted by publication date and was performed up to 23 October 2024 (Beijing time). We employed a manual search strategy using the terms “ginkgo biloba” and “pulmonary fibrosis” along with their variants. The detailed search strategies for each database are provided in Supplementary Material 1.

### 2.2 Eligibility criteria

#### 2.2.1 Inclusion criteria

(1) Participants: Patients with a clinical diagnosis of IPF were considered regardless of nationality, race, gender, occupation, or educational background.

Although the causes of IPF are not limited, all patients should be diagnosed with PF according to at least one of the current or past PF definitions or guidelines, such as:

① Guidelines (Draft) for the Diagnosis and Treatment of Idiopathic Pulmonary Fibrosis (IPF) (Interstitial Lung Diseases Group of the Chinese Medical Association, 2002).
② Consensus on the Diagnosis and Treatment of Idiopathic Pulmonary Fibrosis (IPF) in China (Interstitial Lung Diseases Group of the Chinese Medical Association, 2016).
(2) Intervention: Studies administering GBE alone or in combination with Prednisone in the experimental group.
(3) Comparision: The control group was only given Prednisone.
(4) Outcomes: Studies reporting primary outcomes such as total effective rate, blood gas analysis, and pulmonary function tests; secondary outcomes including inflammatory mediators and fibrosis markers.
(5) Study design: Randomized controlled trials (RCTs) involving patients diagnosed with IPF.

#### 2.2.2 Exclusion criteria

(1) Reviews, case reports, research protocols or conference papers.
(2) Animal and *in vitro* studies.

Two reviewers Xuxin Sun and Ling Peng independently screened the literature according to the above criteria, and the different opinions encountered during the research screening process were resolved through discussion or by the third reviewer Sheng Chen.

### 2.3 Data extraction and quality assessment

Two reviewers (Xuxin Sun and Ling Peng) independently extracted data from the included studies, including first author, publication year, country, intervention and control measures, duration of treatment, demographic information, outcome measures. The risk of bias in the included studies was independently assessed by two reviewers (Xuxin Sun and Ling Peng) using the Cochrane Bias Risk Assessment Tool (RoB2.0), evaluating five aspects of the included RCTs: bias arising from randomization, deviations from established interventions, missing outcome data, outcome measurement, and selective reporting of results. Each aspect was rated as “low risk,” “high risk,” or “possibly risky.” Divergent assessments were resolved through discussion or by a third investigator, and the results are presented in a bias risk map.

### 2.4 Statistical analysis

We performed a meta-analysis using Review Manager 5.4.1. For continuous data, when using the same scale, weighted mean differences (WMD) were calculated, and 95% confidence intervals (CI) were reported. For binary categorical variables, the risk ratio (RR) was used as the effect index for meta-analysis. Heterogeneity tests were based on the p-value obtained from Q tests combined with the I^2^ statistic. The I^2^ statistic is an important indicator of heterogeneity, with values of 25%, 50%, and 75% representing low, medium, and high heterogeneity, respectively. Given the potential for heterogeneity among the included studies in terms of dosages, treatment durations, and study designs, a random effects model (DerSimonian-Laird method) was used throughout the analysis to account for this variability. Subgroup analysis and regression analysis were conducted based on efficacy evaluation criteria and treatment duration to determine the magnitude and source of heterogeneity among studies. Sensitivity analysis was used to evaluate the robustness of the meta-analysis results. Funnel plots were created to assess whether publication bias existed in the included literature, and Egger or Begg methods were used for statistical testing (the number of studies should be ≥5). For results with significant publication bias, the trim and fill method was used to measure the impact of publication bias on the results.

## 3 Results

### 3.1 Literature screening results and flow charts

**FIGURE 1:**
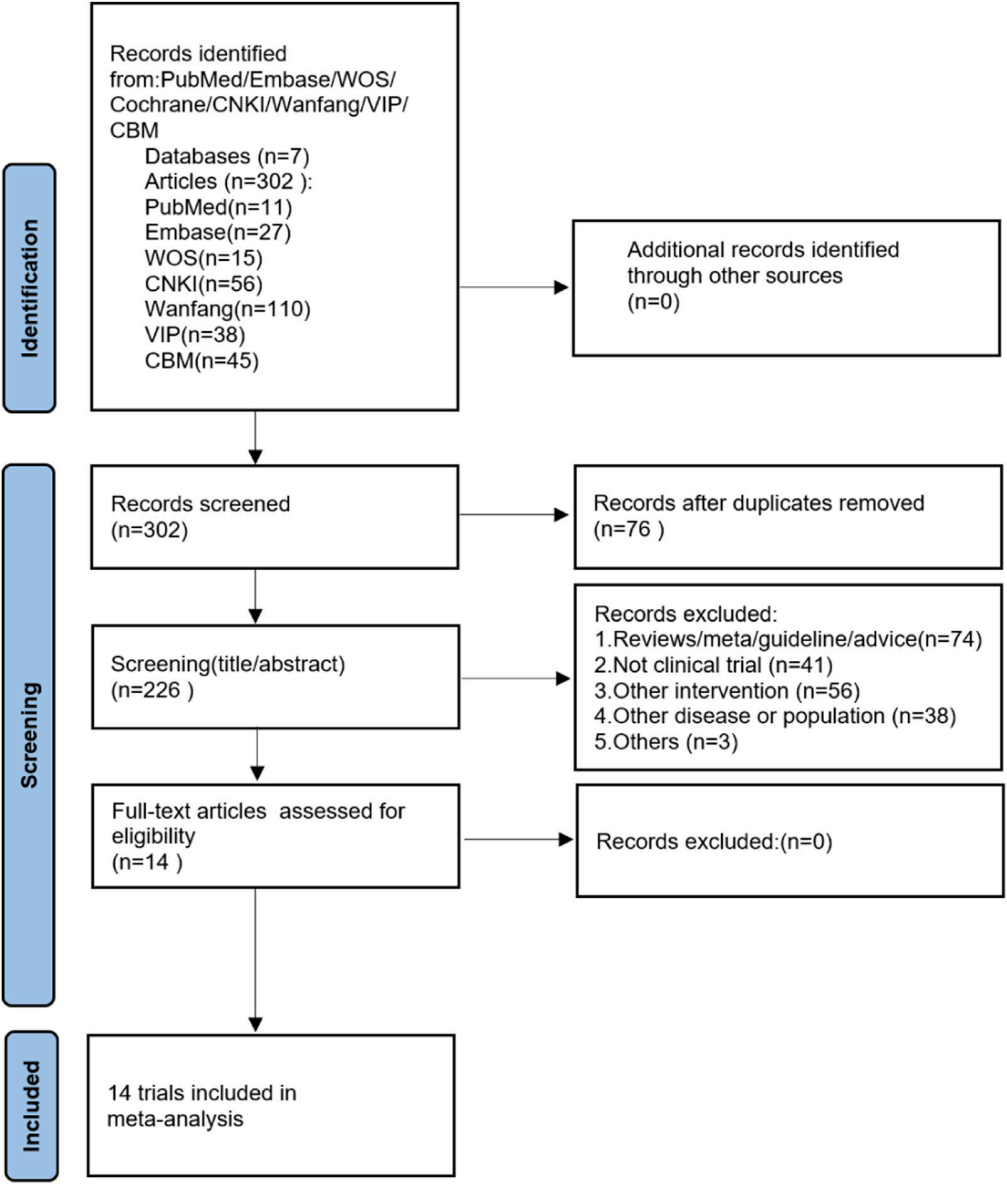
The PRISMA flowchart of the literature search and selection.

A total of 302 papers were retrieved from the initial database search, and no additional studies were identified from the reference scan. After removing duplicates, 226 articles were examined by title and abstract. Of these, 211 articles were excluded because they did not meet the inclusion criteria, 14 articles have been carefully reviewed for full text. Finally, 14 studies were included in this meta-analysis (He et al., 2005; Hao, 2006; Xing et al., 2012; Guan, 2015; Guo, 2015; Li, 2015; Shi, 2016a; Shi, 2016b; Pan and Liu, 2017; Yang and Zhang, 2017; Xu and Liang, 2018; Zhang, 2018; Zou, 2020; Liu et al., 2022b). Figure 1 shows the literature screening process.

### 3.2 Characteristics of included studies

The basic characteristics of the included studies are summarized in Table 1, including first author, publication year, country, intervention and control measures, duration of treatment, demographic information, outcome measures. These fourteen studies encompassed 1043 patients from China, with ages ranging from 42.12 ± 1.25 to 58.60 ± 4.43 years. The experimental group comprised 526 participants, while the control group included 517 participants, with studies published between 2005 and 2022. The sample size across studies varied from 30 to 136 cases. The reported outcome indicators included total effective rate (n = 4), partial pressure of oxygen (PaO2) (n = 8), partial pressure of carbon dioxide (PaCO2) (n = 4), oxygen saturation (SaO2) (n = 3), diffusing capacity of the lungs for carbon monoxide (Dlco) (n = 10), vital capacity (VC) (n = 8), total lung capacity (TLC) (n = 9), forced vital capacity (FVC) (n = 2), forced expiratory volume in one second to forced vital capacity ratio (FEV1/FVC) (n = 2), maximum voluntary ventilation (MVV) (n = 2), 6-min walk test (6MWT) (n = 2), interleukin-4 (IL-4) (n = 4), tumor necrosis factor-alpha (TNF-α) (n = 7), interferon-gamma (INF-γ) (n = 4), hyaluronan (HA) (n = 3), collagen type III (ColⅢ) (n = 2), procollagen type III (PCⅢ) (n = 5), and laminin (LN) (n = 5). Additionally, the treatment duration ranged from 2 to 6 months.

**TABLE 1:** Basic Characteristics of the included studies.

References	Sample size		Country	Male/female		Age(y)		Intervening measure		Main outcome	Treatment course
	E	C		E	C	E	C	E	C		
He et al. (2005)	30	15	China	11/19	7/8	53.96 ± 14.47	47.33 ± 12.50	GBE (Bailuda^®^): 1 g, orally, 3 times daily	PDN: 30 mg, once daily	①②⑤⑥⑦⑬	3 months
Hao (2006)	15	15	China	8/7	7/8	54.17 ± 13.33	52.00 ± 11.09	GBE (Bailuda^®^): 1 g, orally, 3 times daily	PDN: 15–30 mg, once daily	①②⑤⑥⑦	6 months
Xing et al. (2012)	32	38	China	22/10	25/13	57.2 ± 5.6	57.3 ± 7. 1	GBE (Hanshen^®^): 0.25 g, orally, 3 times daily; PDN: 0.125–0.5 mg/kg, once daily	PDN: 0.125–0.5 mg/kg, once daily	⑬	2 months
Guan (2015)	30	30	China	17/13	16/14	49.6 ± 8.7	52.5 ± 9.1	GBE (Tapning^®^): 0.25 g, orally, 3 times daily; PDN: 15–30 mg, once daily	PDN: 15–30 mg, once daily	①⑬	3 months
Guo (2015)	68	68	China	24/44	36/42	53.87 ± 2.09	52.34 ± 2.19	GBE (Tapning^®^): 1g, orally, 3 times daily	PDN: 30mg, once daily	②⑤⑥⑦	3 months
Li (2015)	45	45	China	20/25	22/23	45.9 ± 3.7	46.2 ± 4.0	GBE (999^®^):1g, orally, 3 times daily	PDN: 30mg, once daily	②⑤⑥⑦	3 months
Shi (2016a)	38	38	China	52/14		53.7 ± 8.4		GBE (Hanshen^®^):1g, orally, 3 times daily; PDN: 0. 125–0.5 mg/kg, once daily	PDN: 0.125–0.5 mg/kg, once daily	②③④⑤⑦⑧⑨⑩	3 months
Shi (2016b)	30	30	China	44/16		51.4 ± 9.1		GBE (Hanshen^®^):1g, orally, 3 times daily; PDN: 0. 125–0.5 mg/kg, once daily	PDN: 0.125–0.5 mg/kg, once daily	⑤⑥⑦⑪⑫⑬⑭⑮⑯⑰⑱	3 months
Pan and Liu (2017)	43	43	China	29/14	27/16	47.9 ± 8.0	48.5 ± 8.2	GBE (Hanshen^®^): 1 g, orally, 3 times daily; PDN: 0. 25–0.5 mg/kg, once daily	PDN: 0.25–0.5 mg/kg, once daily	②③④⑤⑧⑨⑩⑰⑱	3 months
Yang and Zhang (2017)	38	38	China	23/15	24/14	56.83 ± 5.77	57.06 ± 6.21	GBE (Tapning^®^): 1 g, orally, 3 times daily; PDN: 0. 125–0.5 mg/kg, once daily	PDN: 0.125–0.5 mg/kg, once daily	⑫⑬⑭⑮⑰⑱	3 months
Xu and Liang (2018)	37	37	China	21/16	21/16	53.29 ± 4.87	53.45 ± 5.24	GBE (Tapning^®^): 1 g, orally, 3 times daily; PDN: 0. 125–0.5 mg/kg, once daily	PDN: 0.125–0.5 mg/kg, once daily	⑤⑥⑦⑪⑫⑬⑭	3 months
Zhang (2018)	40	40	China	25/15	24/16	51.6 ± 7.2	51.7 ± 7.3	GBE (Hanshen^®^):1g, orally, 3 times daily; PDN: 0. 25–0.5 mg/kg, once daily	PDN: 0.25–0.5 mg/kg, once daily	⑤⑥⑦⑫⑬⑭⑮⑯⑰⑱	3 months
Zou (2020)	40	40	China	25/15	24/16	58.60 ± 4.43	58.47 ± 4.36	GBE (Ginaton^®^): 80 mg, orally, 3 times daily; PDN: 0. 125–0.5 mg/kg, once daily	PDN: 0.125–0.5 mg/kg, once daily	②③⑰⑱	3 months
Liu et al. (2022a)	40	40	China	25/15	23/17	45.12 ± 1.15	42.12 ± 1.25	GBE (Yuanzhitong^®^):1g, orally, 3 times daily; PDN:10mg, 3 times daily	PDN: 10mg, 3 times daily	①②③④⑤⑥⑦	3 months

①Total effective rate ②PaO2 ③PaCO2 ④SaO2 ⑤Dlco ⑥VC ⑦TLC ⑧FVC ⑨FEV1/FVC ⑩MVV ⑪6MWT ⑫IL-4 ⑬TNF-α ⑭INF-γ ⑮HA ⑯Col Ⅲ ⑰PC Ⅲ ⑱LN; GBE: ginkgo biloba extract; PDN: prednisone; E: experimental group; C: control group. Bailuda^®^ (Shanghai Sine Promod Pharmaceutical Co., Ltd.; China National Drug Approval No. Z20010169). Hanshen^®^ (Hunan Hanshen Pharmaceutical Co., Ltd.; China National Drug Approval No. Z20026289). Tapning^®^ (Hangzhou Conba Pharmaceutical Co., Ltd.; China National Drug Approval No. Z20063069). 999^®^ (Huangshi Sanjiu Pharmaceutical Co., Ltd.; China National Drug Approval No. Z20040104). Yuanzhitong^®^ (Wuhu Luye Pharmaceutical Co., Ltd.; China National Drug Approval No. Z20040097). Ginaton^®^ (Dr. Willmar Schwabe GmbH & Co. KG; Import Drug Registration No. HC20090014).

### 3.3 Quality assessment

**FIGURE 2:**
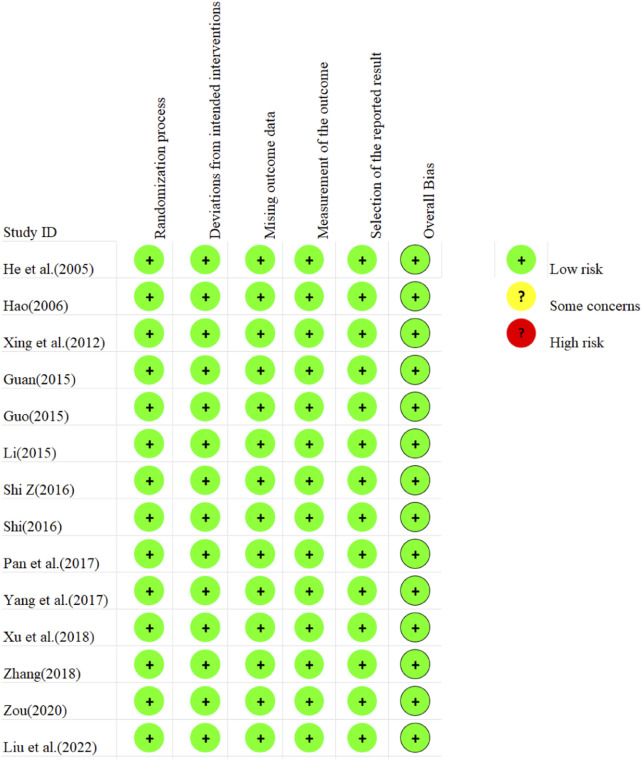
Assessment of risk of bias in the included studies (RCTs).

The risk of bias assessment for the 14 included studies is presented in Figure 2. Regarding bias arising from randomization, all included studies demonstrated low risk due to proper randomization processes. For deviations from established interventions, all studies were assessed as low risk after employing reasonable analytical methods. Similarly, low risk was observed in missing outcome data and outcome measurement across all studies. However, the potential for selective reporting was unclear in all studies, indicating a possible risk of bias. Collectively, the risk of bias within the included literature was small.

### 3.4 Results of meta-analysis

The results of the meta-analysis, along with key heterogeneity statistics and overall effect estimates, are presented in Table 2.

**TABLE 2:** The results of the meta-analysis.

Outcome	Tau^2^	Chi^2^	df	p	I^2^	Z	P
Total effective rate	0.00	0.57	3	0.90	0	3.22	0.001
Arterial blood gas analysis							
PaO2	14.73	35.34	7	<0.001	80	4.30	<0.001
PaCO2	4.23	12.38	3	0.006	76	2.42	0.02
SaO2	0.27	2.48	2	0.29	19	2.27	0.007
Pulmonary function tests							
Dlco (%)	3.03	5.90	5	0.32	15	0.46	0.65
Dlco (mL)	37.32	228.32	3	<0.001	99	0.77	0.44
VC	0.00	2.77	5	0.73	0.0	1.73	0.08
TLC	8.50	7.57	5	0.18	34	0.37	0.71
FVC	0.00	0.03	1	0.87	0	3.04	0.002
FEV1/FVC	0.00	0.54	1	0.46	0	6.90	<0.001
MVV	0.00	0.07	1	0.79	0	6.43	<0.001
6MWT	20.71	1.14	1	0.29	12	6.32	<0.001
Inflammatory factors							
IL-4	3.55	37.28	1	<0.001	97	2.71	0.007
TNF-α(Alveolar Lavage)	10.11	17.55	1	<0.001	94	0.64	0.52
TNF-α	15.31	28.15	2	<0.001	93	1.82	0.07
IFN-γ	2.84	9.34	1	0.002	89	6.32	<0.001
Fibrosis markers							
HA	129.79	7.23	2	0.03	72	4.55	<0.001
Col III	931.70	34.33	1	<0.001	97	0.57	0.57
PC III	233.34	64.03	3	<0.001	95	1.18	0.24
LN	10.87	8.02	4	0.09	50	10.12	<0.001

#### 3.4.1 Total effective rate

**FIGURE 3:**
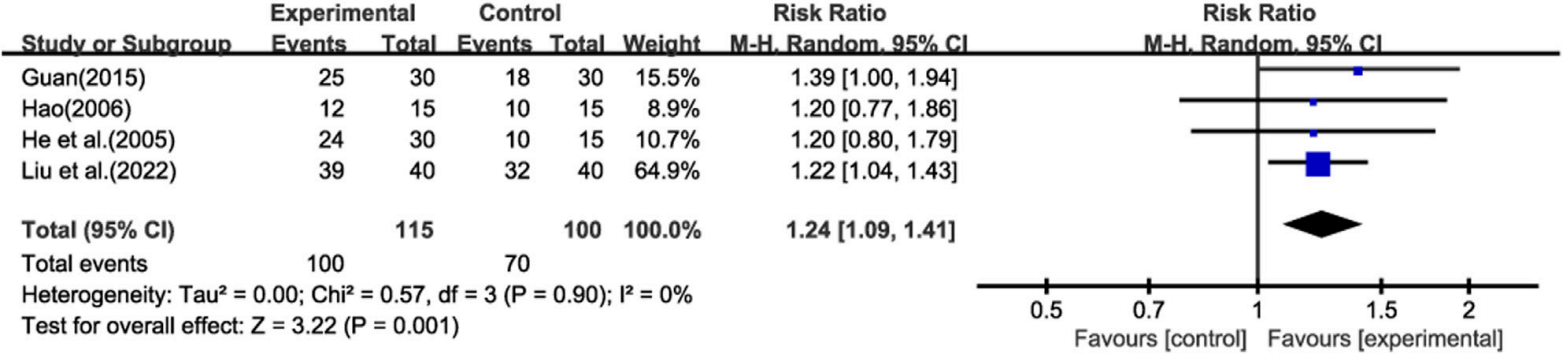
Forest plot of total effective rate.

Four studies (He et al., 2005; Hao, 2006; Guan, 2015; Liu et al., 2022b) reported total effective rate, and meta-analysis was performed using a random-effect model (Figure 3). The results showed that compared with prednisone alone, GBE could improve the total effective rate (RR = 1.24; CI: 1.09 to 1.41; *p* = 0.001).

#### 3.4.2 Arterial blood gas analysis

**FIGURE 4:**
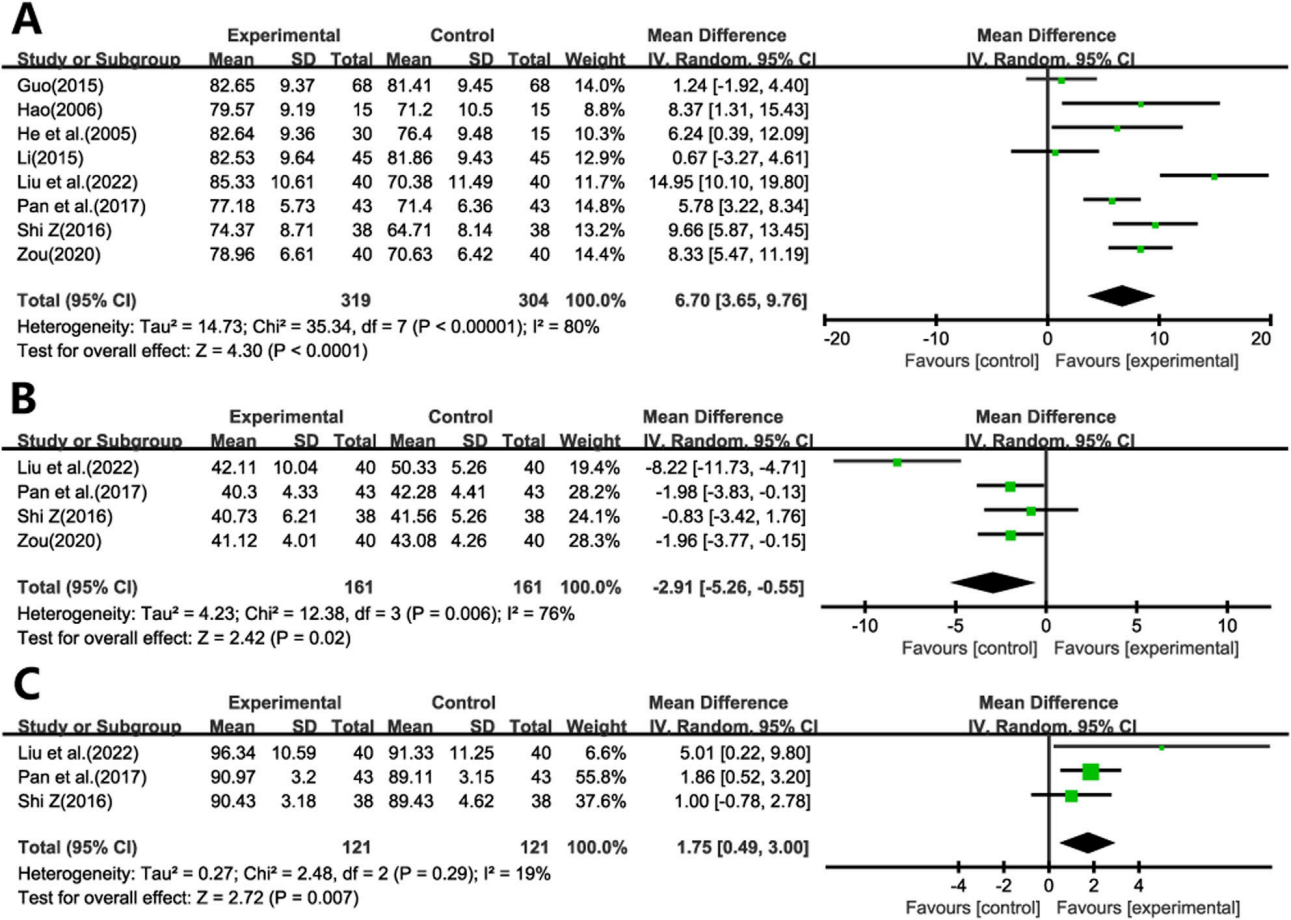
Forest plot of **(A)** PaO2, **(B)** PaCO2, **(C)** SaO2.

Eight studies (He et al., 2005; Hao, 2006; Guo, 2015; Li, 2015; Shi, 2016a; Pan and Liu, 2017; Zou, 2020; Liu et al., 2022b) reported PaO2 (Figure 4A), and four studies (Shi, 2016a; Pan and Liu, 2017; Zou, 2020; Liu et al., 2022b) reported PaCO2 (Figure 4B), the random-effects model demonstrated a significant increase in PaO2 and a decrease in PaCO2 following treatment with GBE, as compared to prednisone monotherapy (WMD = 6.70; 95% CI: 3.65 to 9.76; *p* < 0.001, WMD = −2.91; 95% CI: -5.26 to −0.55; *p* = 0.02, respectively).

Three studies (Shi, 2016a; Pan and Liu, 2017; Liu et al., 2022b) reported SaO2 (Figure 4C), and the pooled analysis indicated that GBE treatment was associated with an improvement in SaO2 levels among patients (WMD = 1.75; 95% CI: 0.49 to 3.00; *p* = 0.007).

#### 3.4.3 Pulmonary function tests

**FIGURE 5:**
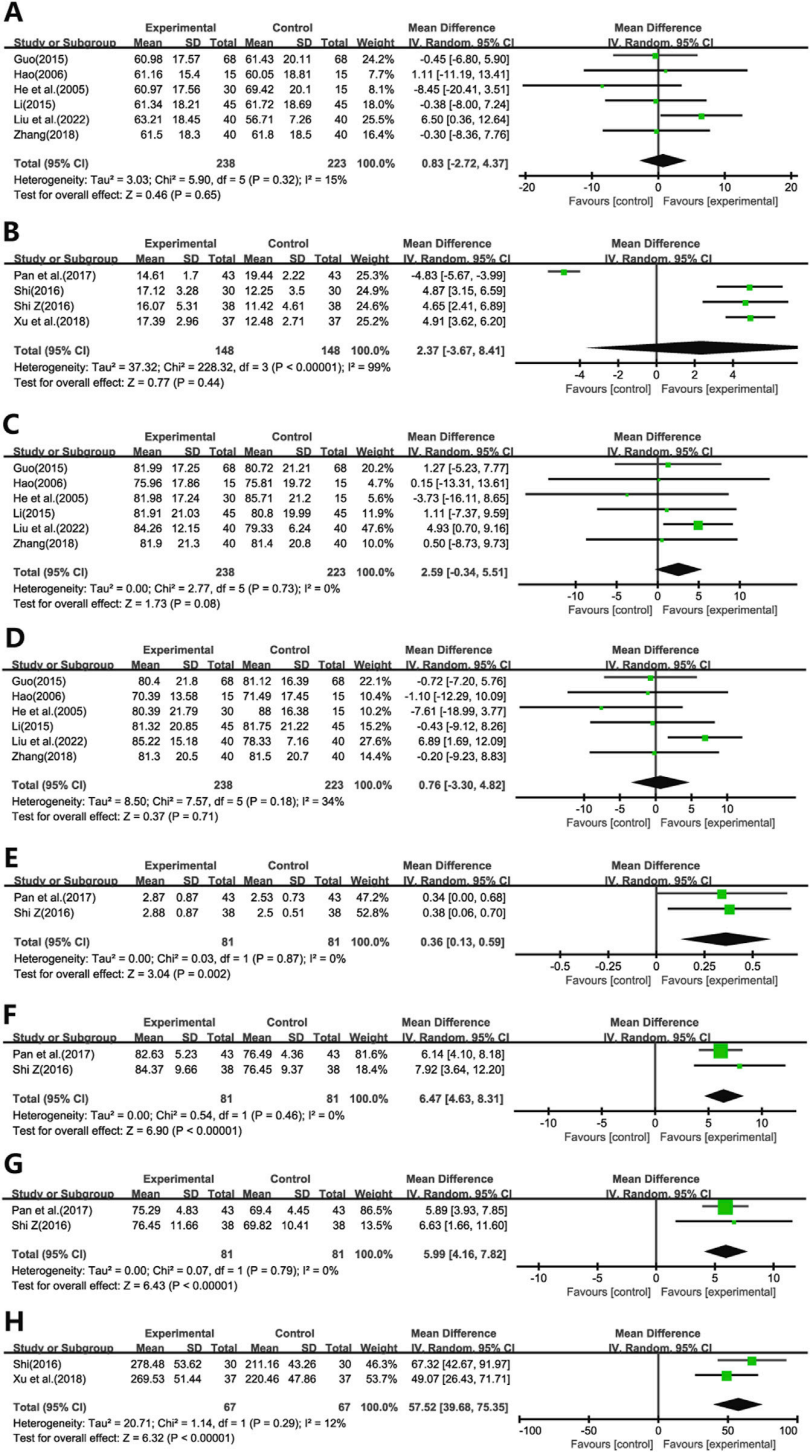
Forest plot of **(A)** Dlco(%), **(B)** Dlco(ml), **(C)** VC, **(D)** TLC, **(E)** FVC, **(F)** FEV1/FVC, **(G)** MVV, **(H)** 6MWT.

Ten studies evaluated the impact of Ginkgo biloba leaf extract on the Dlco in patients with pulmonary fibrosis. Six of these studies (He et al., 2005; Hao, 2006; Guo, 2015; Li, 2015; Zhang, 2018; Liu et al., 2022b) utilized the percentage change (%) as a measure (Figure 5A), while four (Shi, 2016a; Shi, 2016b; Pan and Liu, 2017; Xu and Liang, 2018) employed the absolute change in milliliters (mL) (Figure 5B). The meta-analysis showed no statistically significant differences in Dlco (WMD = 0.83; 95% CI: -2.72 to 4.37; *p* = 0.65, WMD = 2.37; 95% CI: -3.67 to 8.41; *p* = 0.44, respectively).

Six studies (He et al., 2005; Hao, 2006; Guo, 2015; Li, 2015; Zhang, 2018; Liu et al., 2022b) assessed the effects on VC (Figure 5C) and TLC (Figure 5D). The meta-analysis demonstrated no statistically significant differences in VC and TLC (WMD = 2.59; 95% CI: -0.34 to 5.51; *p* = 0.08, WMD = 0.76; 95% CI: -3.30 to 4.82; *p* = 0.71, respectively).

Two studies (Shi, 2016a; Pan and Liu, 2017) evaluated the impact on FVC (Figure 5E), FEV1)/FVC (Figure 5F), and MVV (Figure 5G). The results indicated significant improvements in these pulmonary function parameters in the experimental group compared to the control group (WMD = 0.36; 95% CI: 0.13 to 0.59; *p* = 0.002, WMD = 6.47; 95% CI: 4.63 to 8.31; *p* < 0.001, WMD = 5.99; 95% CI: 4.16 to 7.82; *p* < 0.001, respectively).

Additionally, two studies (Shi, 2016a; Xu and Liang, 2018) assessed the effect on 6MWT (Figure 5H). The findings revealed significant improvements in 6MWT performance in the treatment group compared to the control group (WMD = 57.52; 95% CI: 39.68 to 75.35; *p* < 0.001).

#### 3.4.4 Inflammatory factors

**FIGURE 6:**
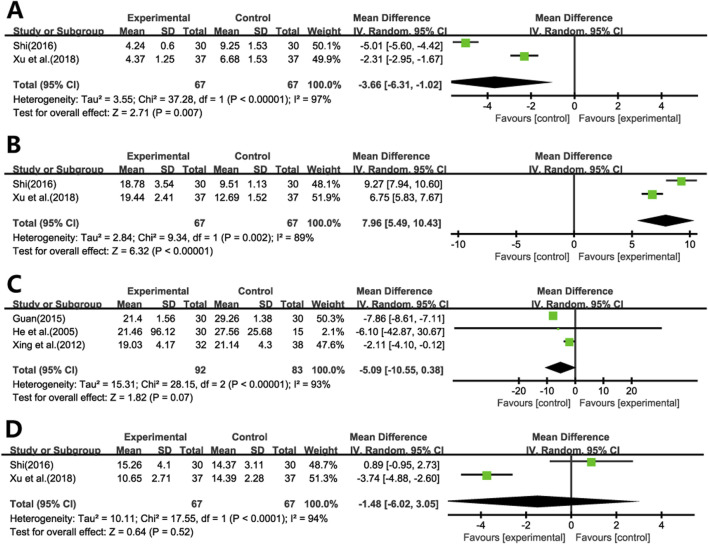
Forest plot of **(A)** IL-4, **(B)** IFN-γ, **(C)** TNF-α, **(D)** TNF-α (Alveolar Lavage).

Two studies (Shi, 2016b; Xu and Liang, 2018) evaluated the effects of Ginkgo biloba leaf extract on IL-4 and IFN-γ levels in patients with pulmonary fibrosis. The results demonstrated significant reductions in IL-4 levels (Figure 6A) and increases in IFN-γ levels (Figure 6B) in the treatment group compared to the control group (WMD = −3.66; 95% CI: -6.31 to −1.02; *p* = 0.007, WMD = 7.96; 95% CI: 5.49 to 10.43; *p* < 0.001, respectively).

Five studies evaluated the impact of Ginkgo biloba leaf extract on TNF-α levels in patients with pulmonary fibrosis, with two studies (Shi, 2016b; Xu and Liang, 2018) utilizing bronchoalveolar lavage fluid specimens (Figure 6C) and three (He et al., 2005; Xing et al., 2012; Guan, 2015) employing blood samples (Figure 6D). The meta-analysis revealed that the differences in TNF-α levels between the treatment and control groups were not statistically significant. (WMD = −1.48; 95% CI: -6.02 to 3.05; *p* = 0.52, WMD = −5.09; 95% CI: -10.55 to 0.38; *p* = 0.07, respectively).

#### 3.4.5 Fibrosis markers

**FIGURE 7:**
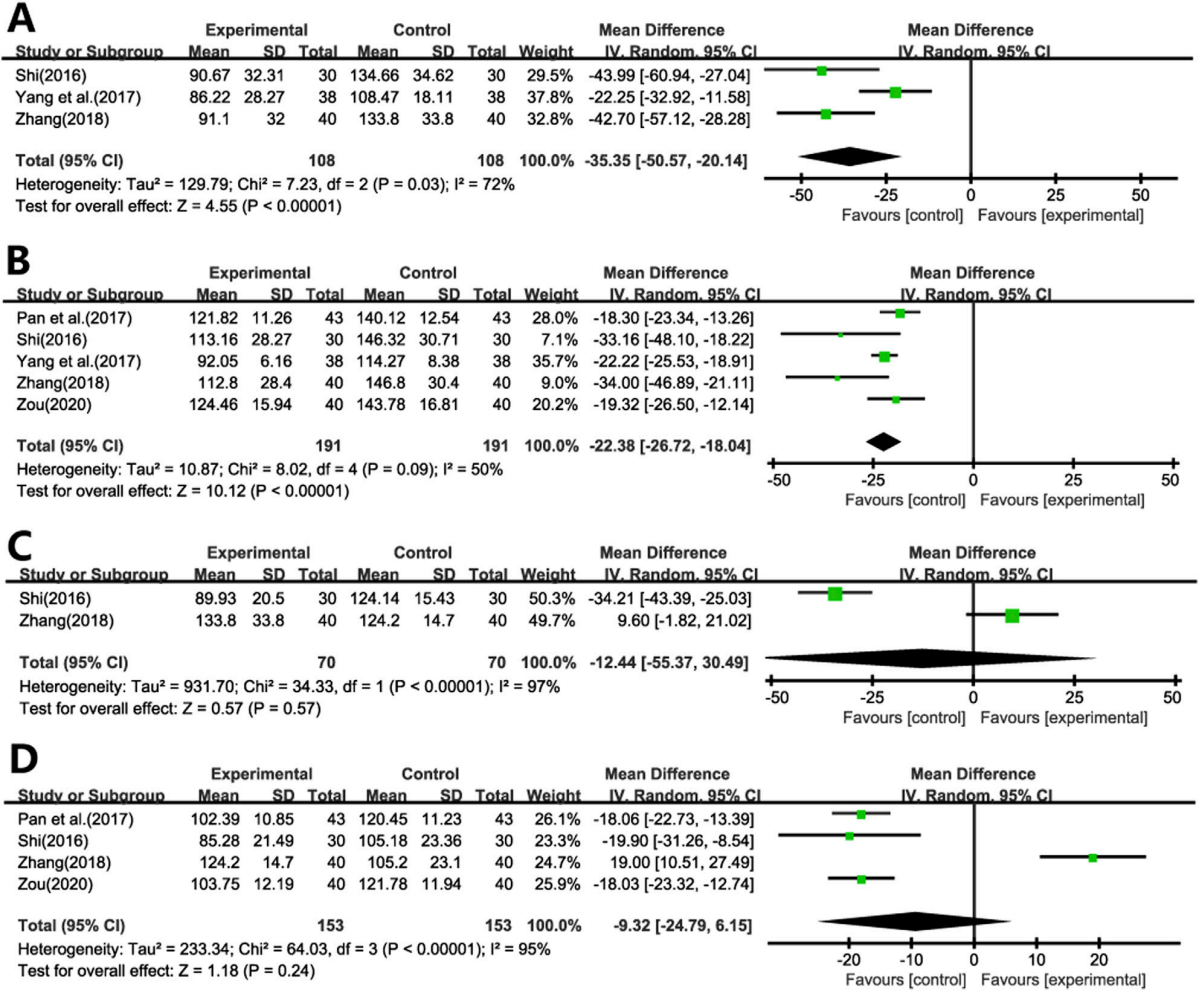
Forest plot of **(A)** HA, **(B)** LN, **(C)** Col III, **(D)** PC III.

Three studies (Shi, 2016b; Yang and Zhang, 2017; Zhang, 2018) evaluated the impact of Ginkgo biloba leaf extract on HA levels in patients with pulmonary fibrosis (Figure 7A). A meta-analysis using a random-effects model indicated significant reductions in HA levels in the treatment group compared to the control group (WMD = −35.35; 95% CI: −50.57 to −20.14; *p* < 0.001).

Five studies (Shi, 2016b; Pan and Liu, 2017; Yang and Zhang, 2017; Zhang, 2018; Zou, 2020) assessed the effects on LN levels (Figure 7B). A meta-analysis using a random-effects model revealed significant reductions in LN levels in the experimental group compared to the control group (WMD = −22.38; 95% CI: −26.72 to −18.04; *p* < 0.001).

Two studies (Shi, 2016b; Zhang, 2018) evaluated the impact of Ginkgo biloba leaf extract on the levels of Col III in patients with pulmonary fibrosis (Figure 7C), while another Four studies (Shi, 2016b; Pan and Liu, 2017; Zhang, 2018; Zou, 2020) assessed the levels of PCIII (Figure 7D). A meta-analysis using a random-effects model indicated no statistically significant differences in both Col III and PCIII levels between the treatment and control group (WMD = −12.44; 95% CI: −55.37 to 30.49; *p* = 0.57, WMD = −9.32; 95% CI: −24.79 to 6.15; *p* = 0.24, respectively).

#### 3.4.6 Adverse events

**FIGURE 8:**
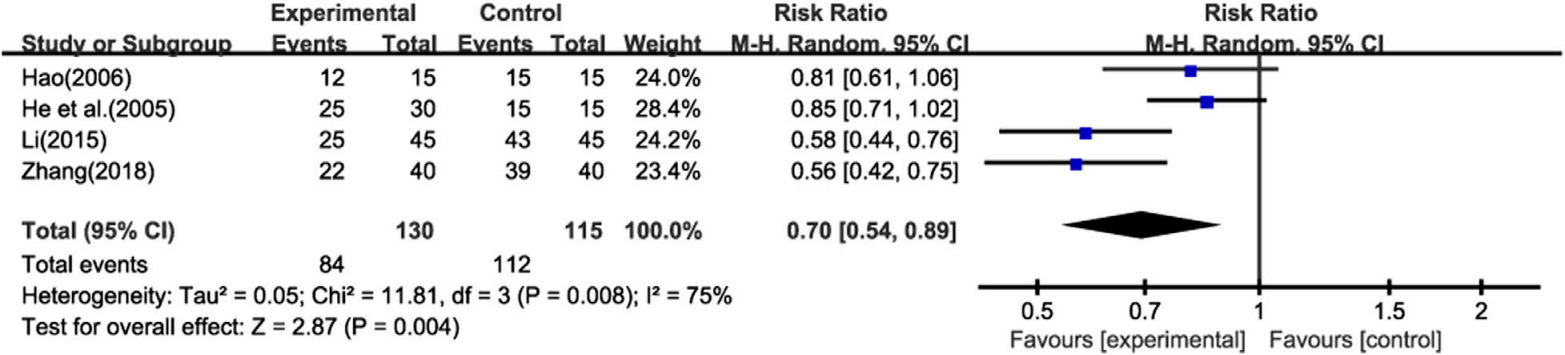
Forest plot of adverse events.

Four studies (He et al., 2005; Hao, 2006; Li, 2015; Zhang, 2018) reported the recurrence of pulmonary infections (≥1 time) during the treatment period. Additionally, none of the studies reported other adverse events (AEs) or serious adverse events (SAEs). This lack of information limits our ability to comprehensively assess the safety of the treatment.Meta - analysis using a random - effects model showed that the recurrence rate of pulmonary infections in the experimental group was lower than that in the control group (Figure 8) (RR = 0.70; 95% CI: 0.54 to 0.89; p < 0.001). However, due to the absence of data on other AEs and SAEs, we are unable to conduct a further analysis on the overall safety of the treatment.

### 3.5 Subgroup analysis

Subgroup analysis of PaO2 based on the use of combination therapy indicated that both Ginkgo biloba extract monotherapy (*p* = 0.033) and its combination with prednisone (*p* < 0.001) significantly increased PaO2 levels compared to the control group.

### 3.6 Sensitivity analysis and publication bias

We have conducted sensitivity analyses for outcome indicators with a study count of three or more. By excluding each study one by one, we have ensured that the results are relatively stable. The sensitivity analysis table is provided in Table 3. To validate the meta-analysis outcomes, we focused on the total effective rate, PaO2, Dlco(%), TLC, VC, and LN as primary outcome indicators, each reported in more than five included studies. Publication bias was quantified employing Begg’s funnel plot and Egger’s linear regression test. The results manifested that total effective rate, PaO2, Dlco(%),TLC, VC, and LN had no significant publication bias (*p* > 0.05).

**TABLE 3:** The results of sensitivity analysis for meta-analysis outcomes.

Outcome	Study removed	Tau^2^	Chi^2^	df	p	I^2^ (%)	Z	P
Total effective rate	He et al. (2005)	0.00	0.57	2	0.75	0	3.09	0.002
	Hao (2006)	0.00	0.56	2	0.76	0	3.11	0.002
	Guan (2015)	0.00	0.01	2	1.00	0	2.67	0.008
	Liu et al. (2022b)	0.00	0.41	2	0.81	0	2.19	0.03
Arterial blood gas analysis								
PaO2	He et al. (2005)	16.33	35.34	6	<0.001	83	3.95	<0.001
	Hao (2006)	15.78	34.97	6	<0.001	83	3.90	<0.001
	Li (2015)	12.56	26.85	6	<0.001	78	4.82	<0.001
	Guo (2015)	11.69	23.99	6	<0.001	75	4.91	<0.001
	Shi (2016a)	15.68	31.73	6	<0.001	81	3.66	<0.001
	Pan and Liu (2017)	20.78	35.20	6	<0.001	83	3.58	<0.001
	Zou (2020)	18.09	32.67	6	<0.001	82	3.54	<0.001
	Liu et al. (2022a)	9.11	21.88	6	<0.001	73	4.03	<0.001
PaCO2	Shi (2016b)	5.59	10.71	2	0.005	81	2.37	0.02
	Pan and Liu (2017)	8.56	12.11	2	0.002	83	1.84	0.07
	Zou (2020)	8.61	12.06	2	0.002	83	1.84	0.07
	Liu et al. (2022b)	0.00	0.60	2	0.74	0	2.95	0.003
SaO2	Shi (2016a)	1.74	1.54	1	0.21	35	1.95	0.05
	Pan and Liu (2017)	4.64	2.37	1	0.12	58	1.24	0.21
	Liu et al. (2022a)	0.00	0.57	1	0.45	0	2.83	0.005
Pulmonary function tests								
Dlco (%)	He et al. (2005)	0.00	3.32	4	0.51	0	1.02	0.31
	Hao (2006)	7.08	5.90	4	0.21	32	0.29	0.77
	Guo (2015)	7.81	5.63	4	0.23	29	0.41	0.68
	Li (2015)	7.62	5.75	4	0.22	30	0.38	0.700
	Zhang (2018)	7.57	5.78	4	0.22	31	0.37	0.71
	Liu et al. (2022b)	0.00	1.69	4	0.79	0	0.55	0.59
Dlco (mL)	Shi (2016b)	40.68	205.84	2	<0.001	99	0.44	0.66
	Shi (2016a)	41.91	184.29	2	<0.001	99	0.41	0.68
	Pan and Liu (2017)	0.00	0.04	2	0.98	0	10.14	<0.001
	Xu and Liang (2018)	41.92	139.27	2	<0.001	99	0.40	0.69
VC	He et al. (2005)	0.00	1.72	4	0.79	0	1.93	0.05
	Hao (2006)	0.00	2.64	4	0.62	0	1.77	0.08
	Guo (2015)	0.00	2.58	4	0.63	0	1.75	0.08
	Li (2015)	0.00	2.64	4	0.62	0	1.75	0.08
	Zhang (2018)	0.00	2.56	4	0.63	0	1.79	0.07
	Liu et al. (2022a)	0.00	0.52	4	0.97	0	0.22	0.83
TLC	He et al. (2005)	3.19	4.85	4	0.30	18	1.06	0.29
	Hao (2006)	12.49	7.33	4	0.12	45	0.33	0.74
	Guo (2015)	13.89	6.93	4	0.14	42	0.33	0.74
	Li (2015)	13.57	7.33	4	0.12	45	0.29	0.78
	Zhang (2018)	13.64	7.40	4	0.12	46	0.26	0.79
	Liu et al. (2022b)	0.00	1.30	4	0.86	0	0.71	0.48
Inflammatory factors								
TNF-α	He et al. (2005)	15.94	28.15	1	<0.001	96	1.76	0.08
	Xing et al. (2012)	0.00	0.01	1	0.93	0	20.67	<0.001
	Guan (2015)	0.00	0.05	1	0.83	0	2.09	0.04
Fibrosis markers								
HA	Shi (2016a)	167.19	4.99	1	0.03	80	3.12	0.002
	Yang and Zhang (2017)	0.00	0.01	1	0.91	0	7.72	<0.001
	Zhang (2018)	184.11	4.53	1	0.03	78	2.97	0.003
PC III	Shi (2016b)	271.44	62.58	2	<0.001	97	0.62	0.53
	Pan and Liu (2017)	462.29	56.55	2	<0.001	96	0.49	0.62
	Zhang (2018)	0.00	0.09	2	0.95	0	10.67	<0.001
	Zou (2020)	456.21	59.14	2	<0.001	97	0.50	0.62
LN	Shi (2016a)	7.73	5.68	3	0.13	47	10.31	<0.001
	Pan and Liu (2017)	14.74	5.77	3	0.12	48	8.52	<0.001
	Yang and Zhang (2017)	30.50	7.76	3	0.05	61	6.48	<0.001
	Zhang (2018)	4.03	4.36	3	0.22	31	11.79	<0.001
	Zou (2020)	16.77	7.56	3	0.06	60	8.31	<0.001

## 4 Discussion

Our study found that GBE significantly improves the total effective rate, PaO2, SaO2, FVC, FEV1/FVC, MVV, 6MWT, IFN-γ, and decreases PaCO2, IL-4, HA, and LN in patients with IPF. These findings underscore the potential therapeutic benefits of GBE in the management of IPF.

The observed improvements in blood gas analysis parameters, particularly the increase in PaO2 and SaO2, suggest that GBE may enhance oxygenation in IPF patients, which is crucial given the impaired gas exchange characteristic of the disease (Otoupalova et al., 2020). This improvement is likely attributable to GBE’s potent antioxidant capabilities, which are believed to mitigate oxidative stress and pulmonary inflammation, consequently facilitating enhanced oxygen diffusion and utilization (Noor et al., 2022). The reduction in PaCO2 levels observed in our study may indicate improved ventilatory efficiency with GBE treatment, which could be particularly beneficial in managing the respiratory acidosis often seen in advanced IPF (Wu et al., 2022). This reduction may also indicate GBE’s beneficial impact on gas exchange and ventilation, potentially mediated through its anti-inflammatory effects, which could alleviate airway inflammation and enhance lung compliance (Tao et al., 2019).

The positive effects on pulmonary function tests, including FVC and FEV1/FVC, indicate that GBE may help to preserve or even improve lung capacity and airflow, which are typically compromised in IPF (Mori and Kondoh, 2021; Wuyts et al., 2020). Previous studies suggest that a ≥5% decline in FVC is recognized as a predictor of mortality in IPF (du Bois et al., 2011), implying that FVC improvement may represent disease stabilization. This preservation or enhancement may stem from GBE’s ability to modulate inflammatory pathways and curtail fibrosis, thereby decelerating the deterioration of lung function (Tian et al., 2018). Furthermore, The improvement in MVV and 6MWT scores further suggests that GBE may enhance the exercise tolerance and overall physical performance of IPF patients, which is closely associated with their quality of life (Mori and Kondoh, 2021; Oğuz et al., 2024). These enhancements are likely due to improved oxygenation, reduced pulmonary vascular resistance, and optimized lung function, all of which contribute to an expanded exercise capacity and elevated physical activity levels.

Inflammation plays a key role in the progression of IPF. In IPF, the Th2-type immune response, characterized by the production of IL-4, is associated with the promotion of fibrosis. IL-4 is known to activate M2 macrophages (Kokubo et al., 2022), which are involved in anti-inflammatory responses, tissue repair, and the deposition of collagen and extracellular matrix (ECM) (Spagnolo et al., 2022). The shift towards a Th2-dominant immune response in IPF is further supported by the increased levels of IL-4 observed in patients, which can lead to tissue inflammation and fibrosis (Heukels et al., 2019). IFN-γ, on the other hand, is a Th1-type cytokine that typically has anti-fibrotic effects. It can inhibit the deposition of collagen by fibroblasts, suggesting that a balance between Th1 and Th2 responses is crucial in the pathogenesis of IPF (Chang et al., 2021; Carvalho et al., 2019).

Recurrent alveolar epithelial injuries triggering the early development of fibrosis (Chambers and Mercer, 2015). These injuries, combined with dysregulated wound repair and myofibroblast dysfunction, lead to sustained tissue remodeling and fibrosis characteristic of IPF (Confalonieri et al., 2022; Younesi et al., 2024). The decrease in IL-4, the fibrosis markers HA and LN, along with the increase in IFN-γ, suggests that GBE may modulate the inflammatory and fibrotic processes in IPF. This modulation is supported by recent research indicating that GBE possesses anti-inflammatory and anti-fibrotic properties (Chummun et al., 2024; Lee et al., 2024), potentially mediated through the regulation of various signaling pathways, including TGF-β1/Smad (Liang et al., 2024).

The anti-inflammatory effects of GBE have been investigated in various studies. Gargouri et al. found that GBE has anti-neuroinflammatory effects in LPS-activated primary microglial cells, which could be relevant to its effects in IPF given the role of inflammation in the disease’s pathogenesis (Gargouri et al., 2018). Additionally, the ability of GBE to modulate cytokine production, as shown by Li et al., who demonstrated the anti-inflammatory effects of GBE components on lipopolysaccharide-stimulated RAW264.7 macrophages, further supports its potential role in reducing inflammation in IPF (Li et al., 2019).

The variability in GBE formulations across studies may influence therapeutic outcomes. As shown in Table 1, extracts varied in dosage forms (e.g., tablets, capsules) and standardization markers. Despite this variability, consistent improvements in oxygenation and fibrosis markers across most studies suggest that core bioactive components (flavonoids and terpene lactones) synergistically drive therapeutic benefits. Future trials should prioritize standardized, high-quality extracts to minimize variability and confirm dose-response relationships.

The potential relevance of our findings extends beyond IPF. Pulmonary fibrosis can also be a serious issue after SARS-CoV-2 infections and may be relevant for post-COVID-19 symptoms. The pathogenesis of post-COVID-19 pulmonary fibrosis involves inflammation and oxidative stress, similar to IPF. Given the anti-inflammatory and antioxidant properties of GBE, it may have therapeutic potential in this context. Several publications have suggested the efficacy of GBE in various symptoms related to SARS-CoV-2 infection (e.g., doi: 10.12659/AJCR.937094). Future research should explore the potential of GBE in treating post-COVID-19 pulmonary fibrosis.

While our findings suggest that GBE could be a valuable adjunct to conventional IPF therapy, it is essential to consider the study limitations. The generalizability of our findings may be limited due to the inclusion of single - center studies conducted exclusively in China, which could affect the applicability of our results to diverse populations. Moreover, the high heterogeneity observed across studies, likely attributable to variations in drug dosage forms, active ingredient content, and demographic characteristics of the study populations, should be taken into account when interpreting the results. In addition, most trials did not report all - cause adverse events or serious adverse events, which restricts the assessment of overall safety. Future studies should adhere to harmonized AE classification systems and prioritize transparency in safety data to address these limitations and provide more comprehensive and reliable evidence for the clinical application of GBE in IPF treatment.

## 5 Conclusion

Our study indicates that GBE may improve clinical outcomes in patients with IPF, including oxygenation, lung function, and exercise tolerance, while modulating inflammation and fibrosis markers. These findings suggest that GBE could be a valuable adjunct therapy for IPF, warranting further investigation in larger clinical trials to confirm its efficacy and safety.

## References

[B1] Carvalho A. É. S. Sousa M. R. R. Alencar-Silva T. Carvalho J. L. Saldanha-Araujo F. (2019). Mesenchymal stem cells immunomodulation: The road to IFN-γ licensing and the path ahead. Cytokine & growth factor Rev. 47, 32–42. 10.1016/j.cytogfr.2019.05.006 31129018

[B2] Chambers R. C. Mercer P. F. (2015). Mechanisms of alveolar epithelial injury, repair, and fibrosis. Ann. Am. Thorac. Soc. 12, S16–S20. 10.1513/AnnalsATS.201410-448MG 25830828 PMC4430974

[B3] Chang C. J. Lin C. F. Lee C. H. Chuang H. C. Shih F. C. Wan S. W. (2021). Overcoming interferon (IFN)-γ resistance ameliorates transforming growth factor (TGF)-β-mediated lung fibroblast-to-myofibroblast transition and bleomycin-induced pulmonary fibrosis. Biochem. Pharmacol. 183, 114356. 10.1016/j.bcp.2020.114356 33285108

[B4] Chummun P. I. Gómez-Llonín A. Bhaw-Luximon A. (2024). From traditional medicine to nanomedicine: potential of Ginkgo biloba extracts in treating inflammatory skin diseases. RSC Med. Chem. 15 (8), 2643–2656. 10.1039/d4md00194j 39149101 PMC11324057

[B5] Confalonieri P. Volpe M. C. Jacob J. Maiocchi S. Salton F. Ruaro B. (2022). Regeneration or repair? The role of alveolar epithelial cells in the pathogenesis of idiopathic pulmonary fibrosis (IPF). Cells 11 (13), 2095. 10.3390/cells11132095 35805179 PMC9266271

[B6] du Bois R. M. Weycker D. Albera C. Bradford W. Z. Costabel U. Kartashov A. (2011). Forced vital capacity in patients with idiopathic pulmonary fibrosis: test properties and minimal clinically important difference. Am. J. Respir. Crit. care Med. 184 (12), 1382–1389. 10.1164/rccm.201105-0840OC 21940789

[B7] Finnerty J. P. Ponnuswamy A. Dutta P. Abdelaziz A. Kamil H. (2021). Efficacy of antifibrotic drugs, nintedanib and pirfenidone, in treatment of progressive pulmonary fibrosis in both idiopathic pulmonary fibrosis (IPF) and non-IPF: a systematic review and meta-analysis. BMC Pulm. Med. 21 (1), 411. 10.1186/s12890-021-01783-1 34895203 PMC8666028

[B8] Flaherty K. R. Wells A. U. Cottin V. Devaraj A. Walsh S. L. F. Inoue Y. (2019). Nintedanib in progressive fibrosing interstitial lung diseases. N. Engl. J. Med. 381 (18), 1718–1727. 10.1056/NEJMoa1908681 31566307

[B9] Gargouri B. Carstensen J. Bhatia H. S. Huell M. Dietz G. P. H. Fiebich B. L. (2018). Anti-neuroinflammatory effects of Ginkgo biloba extract EGb761 in LPS-activated primary microglial cells. Phytomedicine Int. J. phytotherapy Phytopharm. 44, 45–55. 10.1016/j.phymed.2018.04.009 29895492

[B10] Guan Y. (2015). Clinical study and Preliminary exploration of the mechanism of ginkgo biloba leaf in the treatment of idiopathic pulmonary fibrosis. New J. Traditional Chin. Med. 47 (4), 78–80. 10.13457/j.cnki.jncm.2015.04.038

[B11] Guo X. (2015). Therapeutic efficacy and pharmacological actions of Ginkgo Biloba extract in idiopathic pulmonary fibrosis. Chin. Hosp. Pharm. 15 (7). 10.14009/j.issn.1672-2124.2015.07.032

[B12] Hao S. (2006). Clinical study of ginkgo biloba extract in the treatment of idiopathic pulmonary fibrosis. Master’s Thesis.

[B13] He M. Zhang X. Wan H. Shi L. Zhong X. Hao S. (2005). Effects of Ginkgo Biloba extract on pulmonary function and blood gas in patients with pulmonary interstitial fibrosis. Chin. J. Clin. Med. 17 (5). 10.16448/j.cjtcm.2005.05.037

[B14] Heukels P. Moor C. C. von der Thüsen J. H. Wijsenbeek M. S. Kool M. (2019). Inflammation and immunity in IPF pathogenesis and treatment. Respir. Med. 147, 79–91. 10.1016/j.rmed.2018.12.015 30704705

[B15] Hu C. Wang Y. Deng Y. Yao J. Min H. Hu J. (2024). Identification and quantification of the antioxidants in Ginkgo biloba leaf. Biomed. Chromatogr. BMC 38 (11), e5980. 10.1002/bmc.5980 39189506

[B16] Interstitial Lung Diseases Group of the Chinese Medical Association (2002). Guidelines for the diagnosis and treatment of idiopathic pulmonary fibrosis. Chin. J. Tuberc. Respir. Dis. (07), 6–8. 10.3969/j.issn.1671-0800.2003.02.043

[B17] Interstitial Lung Diseases Group of the Chinese Medical Association (2016). Chinese expert consensus on the diagnosis and treatment of idiopathic pulmonary fibrosis. Chin. J. Tuberc. Respir. Dis. 39 (6), 427–432. 10.3760/cma.j.issn.1001-0939.2016.06.005

[B18] Kokubo K. Onodera A. Kiuchi M. Tsuji K. Hirahara K. Nakayama T. (2022). Conventional and pathogenic Th2 cells in inflammation, tissue repair, and fibrosis. Front. Immunol. 13, 945063. 10.3389/fimmu.2022.945063 36016937 PMC9395650

[B19] Kulić Ž. Lehner M. D. Dietz G. P. H. (2022). Ginkgo biloba leaf extract EGb 761(®) as a paragon of the product by process concept. Front. Pharmacol. 13, 1007746. 10.3389/fphar.2022.1007746 36304165 PMC9593214

[B20] Lee B. Roh J. S. Jeong H. Kim Y. Lee J. Yun C. (2024). Ginkgo biloba extract ameliorates skin fibrosis in a bleomycin-induced mouse model of systemic sclerosis. Anim. Cells Syst. Seoul. 28 (1), 152–160. 10.1080/19768354.2024.2337761 38645438 PMC11028018

[B21] Li L. (2015). Clinical analysis of ginkgo biloba extract in the treatment of pulmonary fibrosis. Med. Equip. 28 (16). 10.3969/j.issn.1002-2376.2015.16.079

[B22] Li M. Li B. Hou Y. Tian Y. Chen L. Liu S. (2019). Anti-inflammatory effects of chemical components from Ginkgo biloba L. male flowers on lipopolysaccharide-stimulated RAW264.7 macrophages. Phytotherapy Res. PTR 33 (4), 989–997. 10.1002/ptr.6292 30693991

[B23] Liang W. Yang H. Pan L. Wei S. Li Z. Zhang P. (2024). Ginkgo biloba extract 50 (GBE50) exerts antifibrotic and antioxidant effects on pulmonary fibrosis in mice by regulating Nrf2 and TGF-β1/Smad pathways. Appl. Biochem. Biotechnol. 196 (8), 4807–4822. 10.1007/s12010-023-04755-9 37971580

[B24] Liu C. Liu C. Cai Y. (2022b). Pharmacological actions and therapeutic efficacy of ginkgo biloba extract combined with prednisone in the treatment of idiopathic pulmonary fibrosis. Syst. Med. 7 (23), 183–186. 10.19368/j.cnki.2096-1782.2022.23.183

[B25] Liu G. Y. Budinger G. R. S. Dematte J. E. (2022a). Advances in the management of idiopathic pulmonary fibrosis and progressive pulmonary fibrosis. BMJ Clin. Res. ed 377, e066354. 10.1136/bmj-2021-066354 36946547

[B26] Liu Q. Wang J. Gu Z. Ouyang T. Gao H. Kan H. (2024a). Comprehensive exploration of the neuroprotective mechanisms of ginkgo biloba leaves in treating neurological disorders. Am. J. Chin. Med. 52 (4), 1053–1086. 10.1142/S0192415X24500435 38904550

[B27] Liu Y. Niu P. Yan J. Ji H. Wang Z. Jin X. (2024b). Efficacy and safety of Ginkgo biloba extract in the treatment of unstable angina pectoris: a systematic review and network meta-analysis. J. Ethnopharmacol. 331, 118297. 10.1016/j.jep.2024.118297 38718890

[B28] Mei Q. Liu Z. Zuo H. Yang Z. Qu J. (2021). Idiopathic pulmonary fibrosis: an update on pathogenesis. Front. Pharmacol. 12, 797292. 10.3389/fphar.2021.797292 35126134 PMC8807692

[B29] Moher D. Shamseer L. Clarke M. Ghersi D. Liberati A. Petticrew M. (2015). Preferred reporting items for systematic review and meta-analysis protocols (PRISMA-P) 2015 statement. Syst. Rev. 4 (1), 1. 10.1186/2046-4053-4-1 25554246 PMC4320440

[B30] Mori Y. Kondoh Y. (2021). What parameters can be used to identify early idiopathic pulmonary fibrosis? Respiratory investigation. Respir. Investig. 59 (1), 53–65. 10.1016/j.resinv.2020.10.008 33277230

[B31] Nie S. Zhang S. Wang Y. Zhu M. Chen X. Wang X. (2024). Extraction, purification, structural characterization, and bioactivities of Ginkgo biloba leave polysaccharides: A review. Int. J. Biol. Macromol. 281 (Pt 1), 136280. 10.1016/j.ijbiomac.2024.136280 39368588

[B32] Noor E. T. Das R. Lami M. S. Chakraborty A. J. Mitra S. Tallei T. E. (2022). Ginkgo biloba: A treasure of functional phytochemicals with multimedicinal applications. Evidence-based complementary Altern. Med. eCAM. 2022, 8288818. 10.1155/2022/8288818 PMC8901348 35265150

[B33] Oğuz M. S. Bingöl Z. Pıhtılı A. Karaca Özer P. Sarıtaş Arslan M. Kılıçaslan Z. (2024). Oxygen saturation recovery after 6-minute walk test in patients with idiopathic pulmonary fibrosis. BMC Pulm. Med. 24 (1), 373. 10.1186/s12890-024-03188-2 39085811 PMC11292883

[B34] Otoupalova E. Smith S. Cheng G. Thannickal V. J. (2020). Oxidative stress in pulmonary fibrosis. Compr. Physiol. 10 (2), 509–547. 10.1002/cphy.c190017 32163196

[B35] Pan Y. Liu H. (2017). Efficacy of ginkgo biloba extract combined with glucocorticoids in the treatment of idiopathic pulmonary fibrosis and its impact on serum TGF-β1, IL-13, SOD, and MDA. Mod. J. Integr. Traditional Chin. West. Med. 26 (21), 2312–2315. 10.3969/j.issn.1008-8849.2017.21.010

[B36] Raghu G. Remy-Jardin M. Richeldi L. Thomson C. C. Inoue Y. Johkoh T. (2022). Idiopathic pulmonary fibrosis (an update) and progressive pulmonary fibrosis in adults: an official ATS/ERS/JRS/ALAT clinical practice guideline. Am. J. Respir. Crit. care Med. 205 (9), e18–e47. 10.1164/rccm.202202-0399ST 35486072 PMC9851481

[B37] Shi Z. (2016a). Effects of ginkgo biloba extract combined with prednisone on arterial blood gas and pulmonary function in patients with idiopathic pulmonary fibrosis. J. Hainan Med. Univ. 22 (18), 2143–2145. 10.13210/j.cnki.jhmu.20160606.003

[B38] Shi Z. (2016b). Effects of Ginkgo Biloba extract combined with prednisone on cytokines in BALF of patients with idiopathic pulmonary fibrosis. J. Hainan Med. Univ. 22 (17). 10.13210/j.cnki.jhmu.20160601.005

[B39] Spagnolo P. Tonelli R. Samarelli A. V. Castelli G. Cocconcelli E. Petrarulo S. (2022). The role of immune response in the pathogenesis of idiopathic pulmonary fibrosis: far beyond the Th1/Th2 imbalance. Expert Opin. Ther. targets 26 (7), 617–631. 10.1080/14728222.2022.2114897 35983984

[B40] Tao Z. Jin W. Ao M. Zhai S. Xu H. Yu L. (2019). Evaluation of the anti-inflammatory properties of the active constituents in Ginkgo biloba for the treatment of pulmonary diseases. Food & Funct. 10 (4), 2209–2220. 10.1039/c8fo02506a 30945705

[B41] Tian J. Liu Y. Liu Y. Chen K. Lyu S. (2018). Ginkgo biloba leaf extract protects against myocardial injury via attenuation of endoplasmic reticulum stress in streptozotocin-induced diabetic ApoE(-/-) mice. Oxid. Med. Cell Longev. 2018, 2370617. 10.1155/2018/2370617 29682154 PMC5845491

[B42] Wu Z. Luo Z. Luo Z. Ge J. Jin J. Cao Z. (2022). Baseline level and reduction in PaCO2 are associated with the treatment effect of long-term home noninvasive positive pressure ventilation in stable hypercapnic patients with COPD: A systematic review and meta-analysis of randomized controlled trials. Int. J. chronic Obstr. Pulm. Dis. 17, 719–733. 10.2147/COPD.S344962 PMC8995153 35418751

[B43] Wuyts W. A. Wijsenbeek M. Bondue B. Bouros D. Bresser P. Robalo Cordeiro C. (2020). Idiopathic pulmonary fibrosis: Best practice in monitoring and managing a relentless fibrotic disease. Int. Rev. Thorac. Dis. 99 (1), 73–82. 10.1159/000504763 PMC6979429 31830755

[B44] Xiao L. Tang J. Tan H. Xie Y. Wang S. Xie L. (2024). Efficacy and safety of ginkgo biloba extract combined with donepezil hydrochloride in the treatment of Chinese patients with vascular dementia: A systematic review meta-analysis. Front. Pharmacol. 15, 1374482. 10.3389/fphar.2024.1374482 39021830 PMC11251972

[B45] Xing B. Ding L. Wang C. Wang B. Lu C. Du S. (2012). Effects of Ginkgo Biloba extract combined with prednisone on plasma TNF-α and IL-10 in patients with idiopathic pulmonary fibrosis. Med. Pharm. J. 24 (01), 30–32. 10.3969/j.issn.2095-140X.2012.01.012

[B46] Xu B. Liang L. (2018). Effects of ginkgo biloba extract combined with prednisone on inflammatory factors and pulmonary function in patients with idiopathic pulmonary fibrosis. Med. Equip. 31 (16). 10.3969/j.issn.1002-2376.2018.16.011

[B47] Yang S. Zhang L. (2017). Effects of ginkgo biloba extract combined with prednisone on cytokines and pulmonary fibrosis in patients with idiopathic pulmonary fibrosis. Chin. J. Gerontology 37 (22), 5644–5646. 10.3969/j.issn.1005-9202.2017.22.073

[B48] Yao Y. Zhao J. Li C. Chen Y. Zhang T. Dong X. (2024). Ginkgo biloba extract safety: Insights from a real-world pharmacovigilance study of FDA adverse event reporting system (FAERS) events. J. Ethnopharmacol. 337 (Pt 3), 119010. 10.1016/j.jep.2024.119010 39476880

[B49] Younesi F. S. Miller A. E. Barker T. H. Rossi F. M. V. Hinz B. (2024). Fibroblast and myofibroblast activation in normal tissue repair and fibrosis. Nat. Rev. Mol. cell Biol. 25 (8), 617–638. 10.1038/s41580-024-00716-0 38589640

[B50] Zhang R. (2018). Effects of ginkgo biloba extract and prednisone on cytokine levels and biochemical indicators in BALF of patients with idiopathic pulmonary fibrosis. Chin. J. Health Eng. 17 (1), 22–24. 10.19937/j.issn.1671-4199.2018.01.007

[B51] Zhu Q. Liu D. (2024). Clinical efficacy and mechanism of Ginkgo biloba extract in the treatment of elderly ischemic cerebrovascular disease. Pak. J. Pharm. Sci. 37 (3), 705–713. 10.36721/PJPS.2024.37.3.REG.705-713.1 39340861

[B52] Zou Y. (2020). Clinical observation of ginkgo biloba extract combined with prednisone in the treatment of idiopathic pulmonary fibrosis. Cap. Food Med. 27 (04), 72. 10.3969/j.issn.1005-8257.2020.04.057

