# Supplementary material for: Efficacy of ginkgo biloba extract in the treatment of idiopathic pulmonary fibrosis: a systematic review and meta-analysis of randomized controlled trials
Source: Front Pharmacol. 2025 Mar 5;16:1524505. doi: 10.3389/fphar.2025.1524505 (PMC11919911; doi:10.3389/fphar.2025.1524505)
Supplement: Supplementary file 3 [file DataSheet1.docx]

**Supplementary Material 1: Literature search strategy**

**1. PubMed**

(((((((((("Pulmonary Fibrosis"[Mesh]) OR (Pulmonary Fibrosis)) OR (Fibroses, Pulmonary)) OR (Fibrosis, Pulmonary)) OR (Pulmonary Fibroses)) OR (Alveolitis, Fibrosing)) OR (Alveolitides, Fibrosing)) OR (Fibrosing Alveolitides)) OR (Fibrosing Alveolitis)) OR (Idiopathic Diffuse Interstitial Pulmonary Fibrosis)) AND ((((((((((((((((((((((((((((((((((((((((((("Ginkgo biloba"[Mesh]) OR (bilobas, Ginkgo)) OR (Ginkgo bilobas)) OR (Ginkgo)) OR (Ginkgos)) OR (Ginko)) OR (Ginkos)) OR (Maidenhair Tree)) OR (Maidenhair Trees)) OR (Tree, Maidenhair)) OR (Trees, Maidenhair)) OR (Gingko biloba)) OR (bilobas, Gingko)) OR (Gingko bilobas)) OR (Gingko)) OR (Gingkos)) OR (Ginkgophyta)) OR (Ginkgophytas)) OR (Ginkgo Extract)) OR (Extract, Ginkgo)) OR (Ginkgo biloba extract)) OR (Ginkgo leaf extract)) OR (Tebofortran)) OR (Tebokan)) OR (Tebonin)) OR (EGb 761)) OR (EGb-761)) OR (EGb761)) OR (GBE 761 ONC)) OR (Rokan)) OR (Tanakan)) OR (GBE 761)) OR (GBE-761)) OR (Ginkgo biloba extract 761)) OR (Ginkgo biloba extract 50)) OR (EGB50 Ginkgo biloba extract)) OR (GBE50 (extract))) OR (Ginkgo biloba extract VR456)) OR (VR456)) OR (Ginkgo biloba extract 501)) OR (EGb 501)) OR (Ginkgo biloba extract 1212)) OR (EGB1212))

**2. Embase**

'lung fibrosis'/exp OR 'fibroses, pulmonary' OR 'fibrosis, pulmonary' OR 'pulmonary fibroses' OR 'alveolitis, fibrosing' OR 'alveolitides, fibrosing' OR 'fibrosing alveolitides' OR 'fibrosing alveolitis' OR 'idiopathic diffuse interstitial pulmonary fibrosis' AND 'ginkgo biloba extract'/exp OR 'ginkgo leaf extract' OR 'tebofortran' OR 'tebokan' OR 'tebonin' OR 'egb 761' OR 'egb-761' OR 'egb761' OR 'gbe 761 onc' OR 'rokan' OR 'tanakan' OR 'gbe 761' OR 'gbe-761' OR 'ginkgo biloba extract 761'

**3. Web of science**

Pulmonary Fibrosis (Topic) or Fibroses, Pulmonary (Topic) or Fibrosis, Pulmonary (Topic) or Pulmonary Fibroses (Topic) or Alveolitis, Fibrosing (Topic) or Alveolitides, Fibrosing (Topic) or Fibrosing Alveolitides (Topic) or Fibrosing Alveolitis (Topic) or Idiopathic Diffuse Interstitial Pulmonary Fibrosis (Topic)

AND Ginkgo biloba extract (Topic) or Ginkgo leaf extract (Topic) or Tebofortran (Topic) or Tebokan (Topic) or Tebonin (Topic) or EGb 761 (Topic) or EGb-761 (Topic) or EGb761 (Topic) or GBE 761 ONC (Topic) or Rokan (Topic) or Tanakan (Topic) or GBE 761 (Topic) or GBE-761 (Topic) or Ginkgo biloba extract 761 (Topic) AND Preprint Citation Index (Exclude – Database)

**Four Chinese databases[the China National Knowledge Infrastructure (CNKI), Wanfang DATA, the Chongqing VIP Database (VIP), China Biomedical Database (CBM,Sinomed)] were manually searched，and here is an example of a CNKI search:**

（主题：银杏叶）AND（主题：肺纤维化）
